# Integrity, use and care of long-lasting insecticidal nets in Kirinyaga County, Kenya

**DOI:** 10.1186/s12889-021-10882-x

**Published:** 2021-05-03

**Authors:** Mary Nyangi, Elizabeth Kigondu, Beatrice Irungu, Margaret Nganga, Anthony Gachanja, Martin Murigi, Ruth Nyangacha, Erastus Muniu, Luna Kamau, Jeremiah Gathirwa

**Affiliations:** 1 Department of Chemistry, Kenyatta University, P.O. Box 43844-00100, Nairobi, Kenya; 2 Centre for Traditional Medicine and Drug Research, Kenya Medical Research Institute, P.O. Box 54840-00200, Nairobi, Kenya; 3 Department of Chemistry, Jomo Kenyatta University of Agriculture and Technology, P.O. Box 62000-00200, Nairobi, Kenya; 4 Centre for Public Health and Research, Kenya Medical Research Institute, P.O. Box 54840-00200, Nairobi, Kenya; 5 Centre for Biotechnology, Research and Development, Kenya Medical Research Institute, P.O. Box 54840-00200, Nairobi, Kenya

**Keywords:** Long-lasting insecticide treated-nets, Integrity, Permethrin, α-Cypermethrin, Median concentration, Mass distribution

## Abstract

**Background:**

Vector control is an essential component in prevention and control of malaria in malaria endemic areas. Insecticide treated nets is one of the standard tools recommended for malaria vector control. The objective of the study was to determine physical integrity and insecticidal potency of long-lasting insecticidal nets (LLINs) used in control of malaria vector in Kirinyaga County, Kenya.

**Method:**

The study targeted households in an area which had received LLINs during mass net distribution in 2016 from Ministry of Health. A total of 420 households were sampled using systematic sampling method, where the household heads consented to participate in the study. A semi-structured questionnaire was administered to assess care and use while physical examination was used to determine integrity.

Chemical concentration was determined by gas chromatography mass spectroscopy (GC-MS). Data analysis was done using Statistical Package for Social Sciences (SPSS) version 19.

**Results:**

After 18 months of use, 96.9% (95% CI: 95.2–98.6%) of the distributed nets were still available. Regarding net utilization, 94.1% of household heads reported sleeping under an LLIN the previous night. After physical examination, 49.9% (95% CI: 43–52.8%) of the bed nets had at least one hole. The median number of holes of any size was 2[interquartile range (IQR) 1–4], and most holes were located on the lower part of the nets, [median 3 (IQR 2–5)]. Only 15% of the nets with holes had been repaired. The median concentration for α-cypermethrin was 7.15 mg/m^2^ (IQR 4.25–15.31) and 0.00 mg/g (IQR 0.00–1.99) for permethrin. Based on pHI, Chi-square test varied significantly with the manufacturer (X _(6, *N* = 389)_ = 29.14, *p* = 0.04). There was no significant difference between nets with different number of washes (X^2^(2) = 4.55, *p* = 0.103).

**Conclusion:**

More than three-quarters of the nets supplied had survived and insecticidal potency was adequate in vector control. Standard procedure for field evaluation of surface insecticidal content available to a mosquito after landing on a net to rest is recommended.

**Supplementary Information:**

The online version contains supplementary material available at 10.1186/s12889-021-10882-x.

## Background

In Africa, malaria continues to be a major public health burden. Effective interventions have been put in place to reduce the spread of the disease. Malaria is responsible for extensive mortality and morbidity especially in children; it drains the work force and diverts resources needed for development of a country [1].

Malaria vector control is an essential component in prevention and elimination of malaria. One of the interventions recommended by the World Health Organisation (WHO) to reduce malaria transmission in high risk communities is prevention of mosquito bites. Long-lasting insecticide treated nets (LLINs) have become an effective tool and their use has been intensified. Bednets have been shown to reduce incidence of uncomplicated malaria cases by 50%, severe malaria by 45% and malaria mortality by 55% [2].

Nearly 28 million Kenyans live in malaria endemic areas [3]. Investment in malaria control over the last 5 years has had a positive impact on the overall malaria related morbidity and mortality cases. The most successful investment on malaria control has been the distribution of LLINs by the national malaria control program (NMCP) and other partners like centre for disease control (CDC).

More than 74% of households in malaria endemic areas own LLINs; majority of which have been distributed free of charge. However, there is little information about the actual durability [4] of the LLINs; with reports of net misuse in Kwale County, Kenya reported [5]. Nets which are intact act as physical barriers which prevent the vector from having contact with human and thus providing personal protection [6]. A bed net offers personal protection provided it is in good physical condition [7].

Protection of bed nets diminishes with increased number of holes regardless of whether the net is treated or not [8]. In order to enhance protection from mosquito bite an insecticide is added to the net fibres [9]. According to Kenya malaria indicator survey (2010), 57% of Kenyan households own at least one net [10].

Hence, follow up of nets used in the field is paramount in assessing the proportion of nets that is able to offer personal protection from mosquito bite. Most studies carried out in Kenya assessed physical state and bio-efficacy of LLINs after distribution but none of the studies have been done in Kirinyaga County. The current study was conducted after mass net distribution had been carried out. It involved the due objectives of; assessing the integrity, use and care of LLINs 18 months after their distribution.

## Methods

### Study area and population

**Fig. 1:**
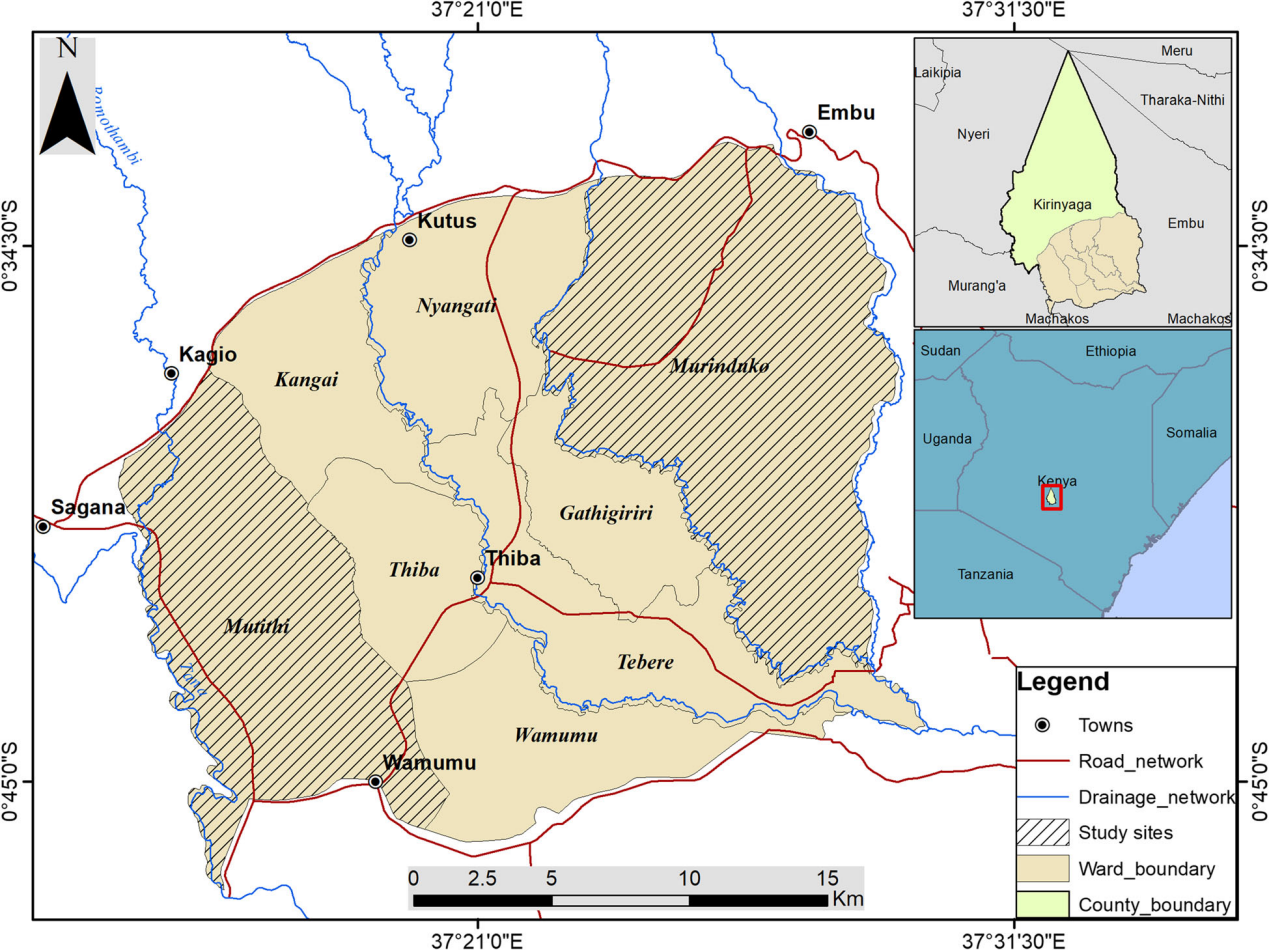
Kirinyaga County showing the study area of Mwea East and Mwea West (the map was generated in ArcGIS Desktop 10.5 software which requires a user license)

The study area was Mwea West and Mwea East Sub-counties in Kirinyaga County, Kenya (0.6591° S, 37.3827° E) approximately 100 km North East of Nairobi at an altitude of about 1159 m above sea level. Kirinyaga County has a population density of 246 persons per km^2^ in a total area of 581km^2^. The County is one of the areas with highest vector populations due to rice irrigations specifically in the Mwea region. Mwea East is predominantly rice growing whereas Mwea West’s main economic activity is horticulture (Fig. 1).

### Study design

The study adopted a cross-sectional household survey that took place between April and May 2018. The study targeted all households where LLINs had been distributed by the Ministry of Health in November 2016 during a mass net campaign. The Kirinyaga County Public Health Office (PHO) was the entry point where the register of households that had received the nets was availed to this study.

The research adopted multistage sampling procedure. Stage one involved selection of two sub-counties namely Mwea East and Mwea West from the five sub-counties of Kirinyaga.

County based on prevailing ecological setting (the two sub-counties have an approximate population of 142,926 people). Stage two was selection of villages from the registered 281 villages in the two sub-counties (Mwea East 141 and Mwea West 140 villages). Villages have an average of 100 households and approximate population of 500 persons. To obtain a sample size of 405 households using a uniform sample take of 15 households per village, 27 villages were required with one extra village added, to cater for missing households or where occupants had relocated to other areas. Proportionate allocation method was used to determine number of villages needed per sub-county and in each sub-county, systematic sampling with a random start was applied to sample required villages. The systematic sampling method was applied as it enables sampled villages across sub-county to be evenly distributed and yield good estimates for the population parameters.

The final stage involved systematic sampling with a random start of eligible households within selected villages (15 households per village).

The inclusion criteria for the study were those nets which were distributed during mass net campaign of November 2016. Any other net found in the homestead and not a 2016 campaign net was excluded from the study.

The field team explained the survey objectives to the household heads before administration of the questionnaire. The field team comprised of researchers and community health workers. The community health workers had been trained on the questionnaire prior to the study. Training content included seeking written informed consent, technique of sampling nets for chemical analysis and classification of hole size.

A total of 420 household heads/spouses consented and participated in the study. Written informed consent was obtained from the household heads (Additional file 1). A questionnaire and physical examination of LLINs were used to collect data (Additional file 2).

The nets were removed from their hanging place and assessed outside the house for the presence of holes. Hole sizes were categorised into four groups; size 1, 2, 3 and 4.

### Assessing LLINs physical and chemical integrity

Integrity of the nets was quantified as described in WHOPES [11]. The area of each ‘hole size’ was calculated from an assumed diameter. Size one hole diameter; 1.25 cm, size two hole diameter; 6 cm, size three hole diameter; 17.5 cm and size four hole diameter; 30 cm [12]. Proportionate hole index (pHI) for each net was calculated by adding the areas of all hole sizes present in a net.

A random sample of 80 nets was withdrawn from the households (with replacement done) for chemical analysis. From each side of the five sides of a net 30 cm × 30 cm piece was cut and the pieces for each net pooled together to ensure there was no bias, wrapped in aluminium foil, labelled and stored in separate bags for transportation to KEMRI laboratories for chemical analysis. Five new and unused LLINs from the same batch as the nets distributed were also provided by the PHO. These new nets were used to develop baseline data for chemical testing.

### Sample preparation

Mechanical extraction was used since only the surface concentration of the insecticide in the netting material is available to an alighting mosquito [13]. A 10 cm × 10 cm piece was cut from the 30 cm × 30 cm sample, weighed and the total mass recorded [14]. The sample was then cut into smaller pieces and introduced in a glass vial equipped with a tight stopper containing 5 ml of analytical grade methanol. The insecticide was extracted from the net by ultra-sonicating at room temperature for 30 min [13]. The extract was filtered through a 0.45 μm polytetrafluoroethylene (PTFE) syringe filter and appropriate dilution was made. Methanol was evaporated from the extract before reconstituting with hexane, to bring down the permethrin and α-cypermethrin concentrations within the concentration range of the standards, of between 20 ppb and 500 ppb.

### Standard preparation

All solvents used for the analysis were high performance liquid chromatography (HPLC) grade. Pesticide standards used were of 99.9% purity. Working standards were prepared on the day of analysis and stock solutions stored at 4 °C at all times. The limit of detection and limit of quantification for the instrument were also determined as part of method development.

### GCMS instrumentation

A Shimadzu QP 2010-SE GCMS coupled to an auto sampler was used for the analysis. Ultrapure Helium was used as the carrier gas at a flow rate of 1 ml/minute. A BPX5 non polar column, 30 m; 0.25 mm ID; 0.25 μm film thickness, was used for separation. The GC was programmed as follows: 50^0^ C (1 min); 30^0^ C /min to 300^0^ C. Only 1 μL of the sample was injected. Injection was done at 200^0^ C in split mode, with split ratio set to 10:1. The interface temperature was set at 280^0^ C. The Electron Ionisation (EI) ion source was set at 200^0^ C. Mass analyses was done in Single Ion Monitoring (SIM) mode at specific retention windows. SIM group ions for permethrin were 127, 163 and 183 m/z; with 183 m/z being the quantifier ion. The retention window for these ions was between 24 and 26 min. SIM group ions for α-cypermethrin were 127, 163 and 181; with 163 m/z being the quantifier ion. The retention window for these ions was between 26.5–28.5 min. To test the method suitability, extraction efficiency (Additional files 3, 4, 5, 6 and 7), repeatability (Additional files 8, 9, and 10), accuracy and limit of detection (Additional file 11), were determined before sample injection. All samples were analysed at the Jomo Kenyatta University of Agriculture and Technology (JKUAT) analytical chemistry laboratory.

### Data analysis

Data collected using questionnaire was entered into a Microsoft excel sheet before being exported to SPSS version 19. For continuous data, distribution characteristics were confirmed using Kolmogorov-Sminorf test and Exploratory Data Analysis (EDA). For continuous variables means, medians and standard deviations were calculated and for categorical data, proportions and 95% Confidence Intervals. Testing for difference between grouping variable categories was performed using Chi-square (for categorical data), Student T test or One-way analysis of variance (ANOVA) (for continuous normally distributed data) and Mann-Whitney U test or Kruskal Wallis test (for continuous skewed data) depending on number of grouping variable categories.

The number of holes in a net were used to calculate the proportionate hole index (pHI). Each hole was weighted by its size and summing them up for each net. WHO formula for pHI was used; pHI = (area/1.23 x no. of size-1 holes) + (area/1.23 x no. of size-2 holes) + (area/1.23 x no. of size-3 holes) + (area/1.23 x no. of size-4 holes).

The area for each net was calculated on the assumption that the holes are circular and the diameter is equal to the midpoint in each hole size category. [Categories; holes smaller than a thumb (0.5–2.0 cm), holes larger than a thumb but smaller than fist (2–10 cm), holes larger than a fist but smaller than a head (10–25 cm), holes larger than a head (> 25 cm)]. A net with pHI of 0–64 is a good net, a net with pHI of between 65 and 642 is a damaged net and a net with a pHI ≥643 is a net which is too torn [12].

### Ethical approval

Approval for this study was sought from the Kenya Medical Research Institute (KEMRI) Scientific and Ethics Review Unit (SERU), approval number KEMRI/SERU/CTMDR/037/3374.

## Results

### Household demographic

Table 1 shows the characteristics of household heads/spouses in Mwea East and Mwea West of Kirinyaga County. Of all the 420 household heads/spouses who consented to participate in the study, 298 (71%) were females and 122 (29%) were males. The median age of respondents was 40 years (IQR: 30 - 51 years) and ranged from 17 to 90 years. Nearly 60.4% of them had no formal education or educated up to primary school level.

**Table 1:** Social demographic characteristics of Household heads/spouse who received LLINs in November 2016

Characteristics	Number ***N***^a^ = 420	%
**Sex of respondent**		
Male	122	29.0
Female	298	71.0
**Level of education**		
Without education	26	6.1
Lower primary	228	54.3
O- Level	141	33.6
College	25	6.0

^a^Number

### Bed net use and care

Of the 420 sampled households, 407 or 96.9% (CI: 95.2–98.6%) of the nets distributed in November 2016 were still present (Table 2). The proportion of nets that were in use the night prior to survey day out of 407 was 94.1%, and on visual observations all the nets were hanged over the bed during the survey. Of the nets present, 24 or 5.9% had not been used the night prior to the survey. Out of those 24 nets that were not in use, 13 were new and still in their original package. Other reasons why the net had not been used; owners had forgotten to hang (*n* = four) net used for other purposes (misused, *n* = four), while three of the nets were reported to have been too torn for use.

**Table 2:** Assessing physical presence, use and care of the LLINs in Kirinyaga County 18 months after distribution

Characteristics	Number	%
Is the net still present?	***N***^a^ **= 420**	
Yes	407	96.9
No	13	3.1
Was the net in use last night?	***N*** **= 407 (Net present)**	
Yes	383	94.1
No	24	5.9
Is the net intact?	***N*** **= 383 (Net used)**	
Yes	192	50.1
No	191	49.9
Manufacturer’s name	***N*** **= 407 (Net present)**	
Manufacturer A	288	70.8
Manufacturer B	87	21.4
Manufacturer C	26	6.4
Not identified	6	1.4

^a^ Number

### Physical and chemical integrity

**Fig. 2:**
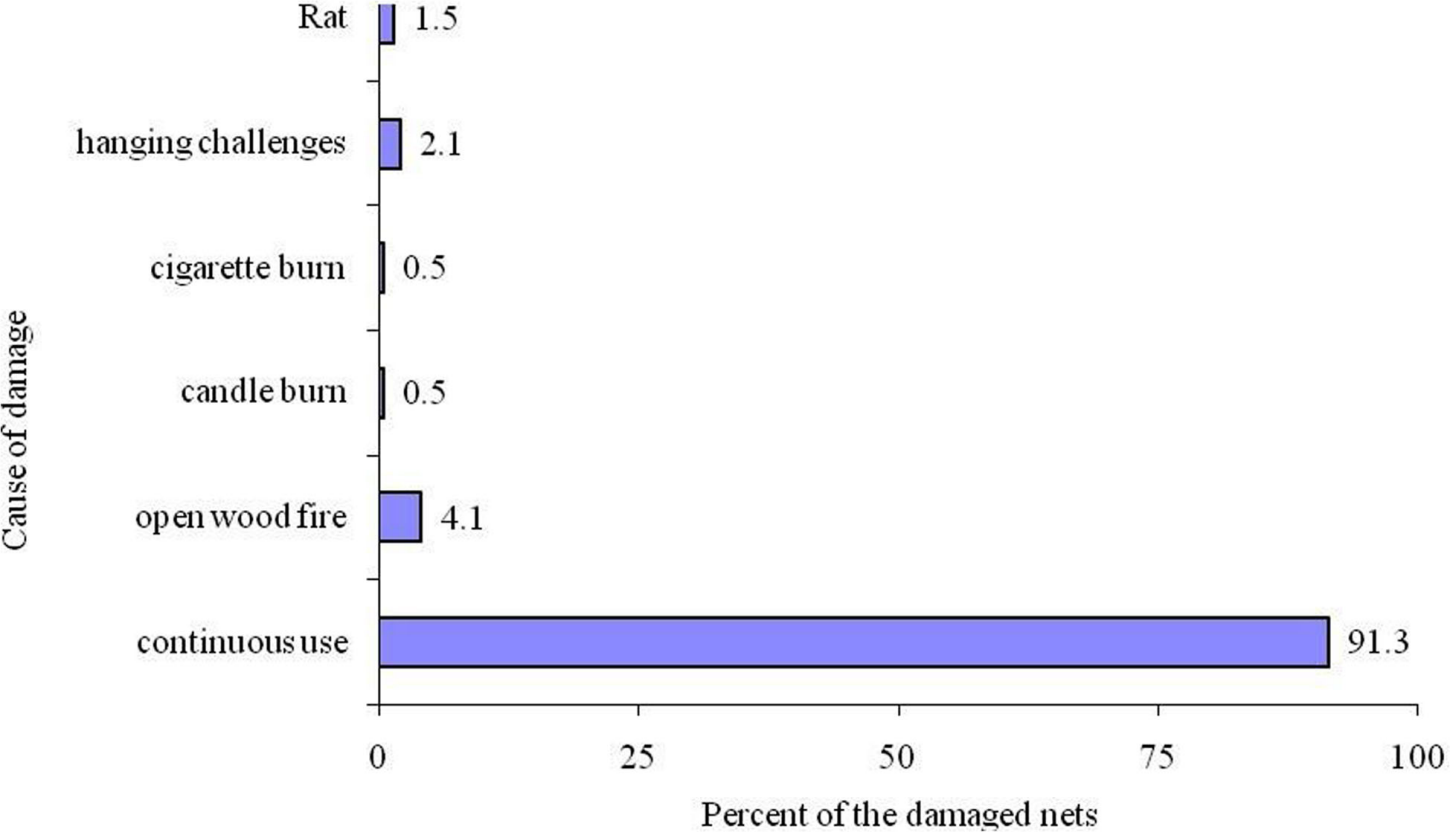
Cause of holes found in LLINs within Kirinyaga County

Almost half of the 383 nets in use (*n* = 191) or 49.9% (95% CI: 43–52.8%) had at least one hole. More than 85% (*n* = 704 out of 820) of the total holes in nets were located on the lower part of the nets [median number of holes 3 (IQR 1–5)]. The four sizes of holes (1, 2, 3 and 4) were present in the nets, of which size two was the most common at 29%. The median number of holes of any size was two [inter-quartile range (IQR) 1–4] (Table 3). A minimum of one hole and a maximum of 18 holes were counted in a net. Reasons for net damage varied from continuous use (*n* = 173 out of 191), open wood fire (nine), candle burn (one), cigarette burn (one), hanging challenges (four) and rodent damage (three) (Fig. 2).

**Table 3:** LLINs holes characteristics [median and inter-quartile range (IQR 25–75)]

Size and location of hole	***N***^b^	Median	IQR^a^	Total hole area (cm^**2**^)	Total hole area quartiles
Size one	239	2.00	1.00–3.00	282	0.00–1.23
Size two	259	2.00	1.00–4.00	88	28.28–1132.12
Size three	168	2.00	1.00–4.00	61	240.56–962.24
Size four	154	2.00	1.00–4.00	60	706.95–2827.80
upper part of net	116	3.00	2.00–5.00		
lower part of net	704	3.00	1.00–5.00		

^a^Interquatile range

^b^Number

All the nets distributed were polyethylene LLINs, of which 294 were from manufacturer A, 87 from manufacturer B while 26 nets were from manufacturers C. Six of the nets had their label washed off and thus the manufacturer could not be identified.

When the nets with holes, were grouped according to the manufacturer, 42% were from manufacturer A, 56% from manufacturer B, and 81% from manufacturer C.

**Fig. 3:**
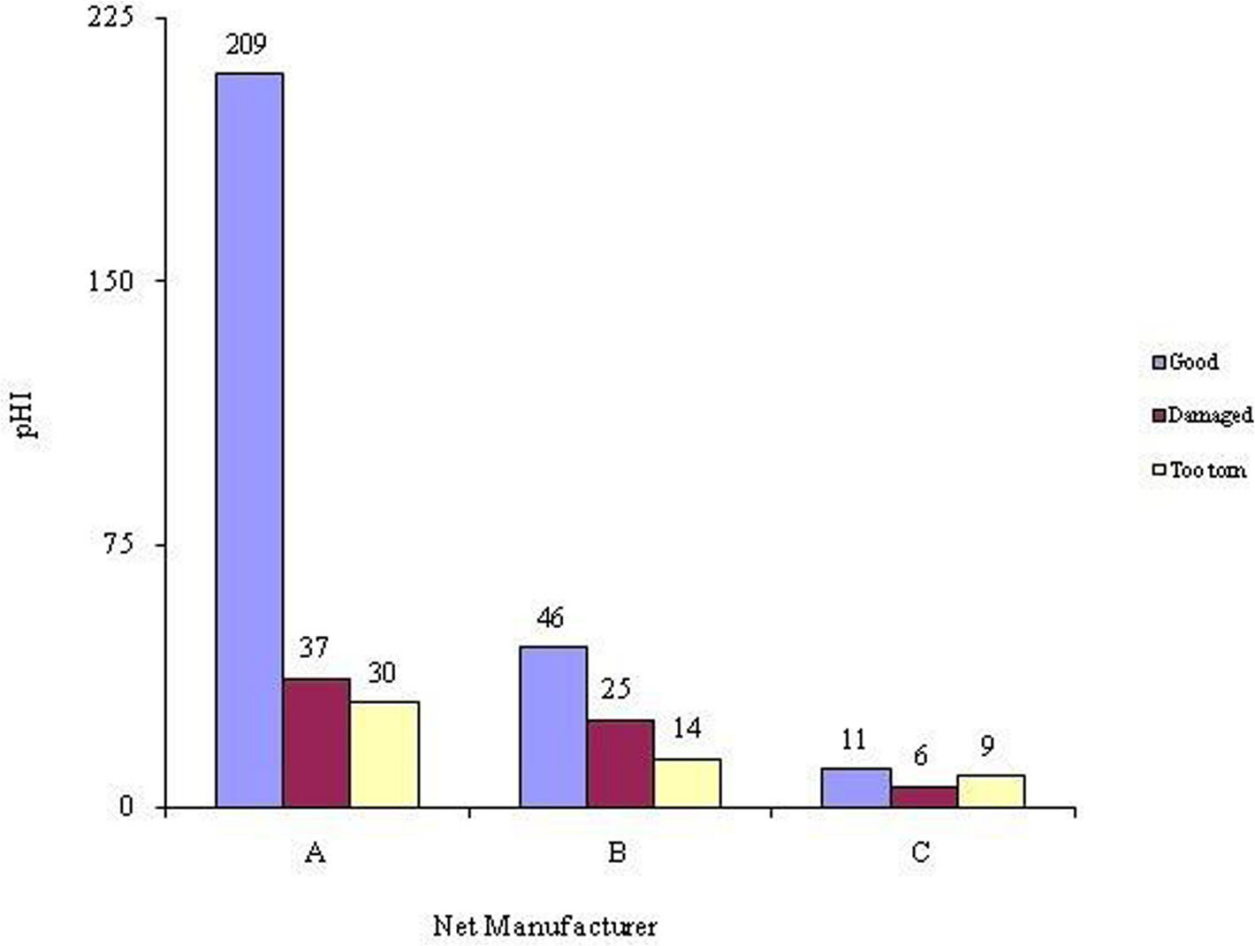
Categories of the LLINs by proportionate hole index

“Good” and “damaged” nets were further categorised as serviceable nets. Manufacturer A had the highest proportion of serviceable nets at 85.4% (*n* = 246 out of 288), manufacturer B 81.6% (*n* = 71 out of 87), manufacturer C had the highest proportion of nets with holes recorded and also the lowest proportion of serviceable nets at 65.3% (*n* = 17 out of 26) (Fig. 3). In general 85.8% (95% CI: 82.3–89.2%) of the nets sampled in the study were serviceable after 18 months of continuous use. pHI ranged from zero to 6480. Based on pHI, Chi-square test varied significantly with the manufacturer (X _(6, *N* = 389)_ = 29.14, *p* = 0.04).

Up to 85% of the nets under routine use had holes, and had not been repaired. Those repaired were either by tying a knot or hand sewing.

When the baseline concentration of the pyrethroid in the nets was compared, the median surface concentration for α-cypermethrin treated nets was 39.4 mg/m^2^ (IQR 39.15–42.10) and 6.98 mg/g (IQR 0.10–17.02) for permethrin treated nets. For the used nets the median for α-cypermethrin and permethrin treated nets was 7.1 mg/m^2^ (IQR 4.25–15.13) and 0.00 mg/g (IQR 0.00–1.99) respectively (Table 4). Insecticide content differed significantly within the nets with some net samples having undetectable insecticidal content (Additional files 12 and 13).

**Table 4:** Median and inter-quartile range (IQR 25–75) of active ingredients in baseline and washed nets

	Active ingredients	***N***^a^	Median	IQR^b^
Baseline nets	permethrin	20	6.98	0.10–17.02
	α-cypermethrin	5	39.400	39.15–42.10
Washed nets	permethrin	40	0.00	0.00–1.99
	α-cypermethrin	40	7.15	4.25–15.31

^a^Number

^b^Interquatile range

When pyrethroid content in a net for both permethrin and α-cypermethrin was compared to the number of washes a net had undergone, the difference was not significant (X^2^(2) = 4.55, *p* = 0.103) for α-cypermethrin treated nets and (X^2^(2) = 3.83, *p* = 0.148) for permethrin treated nets.

## Discussion

This study assessed the integrity (physical and chemical), use and care of bed nets distributed within two sub-counties of Kirinyaga County, Kenya, eighteen months after mass net distribution campaign of 2016. This study is novel because no other study has assessed the status of bed nets after their distribution in Kirinyaga County which is one of the malaria endemic areas in Kenya mostly due to rice irrigated farms.

High net usage in the study area is in line with earlier reports of net utilisation [15, 16]. But this rate of net usage has been found to decrease as the nets get older and the number of holes increases [17]. The high net usage in the area could be due to risk perception of the rice irrigation farms which ensure larva development of the mosquito vector all year round. The continuous use of bed net and increased availability of antimalarials has seen a speedy drop in malaria incidences in Mwea, according to unpublished report by department of Health, Kirinyaga County. The success of reduced malaria cases could also be attributed to improved irrigation methods and behaviour change where people have accepted use of free nets [18, 19]. To an extent, due to the prolonged dry season experienced in the study area for the last 2 years resulting in reduced rice farming acreage.

The small number of nets unable to offer personal protection (as reported by respondent) changed from three to twenty seven when the nets surveyed were classified according to WHO criteria of serviceable and too torn nets. Classifying nets as serviceable nets (good and damaged) shows the ability of a net to inhibit mosquito bite even when they are in “damaged” state. This partly, could be attributed to the repellent effect of the pyrethroid incorporated or coated in the nets [20]. This is unlike what had been found in Uganda where considerable physical net damage had occurred within 1 year of bed net use [21, 22]. This low number of torn nets in the current study could be accredited to the sensitization campaign carried out by the National Malaria Control Program (NMCP) through the Ministry of Health, on use, care and maintenance of bed nets, carried prior to net distribution [10, 20]. Education information messages on net maintenance were seen to improve on net deterioration as reported by Spencer and others after a pre-distribution education campaign in South West Uganda [22]. The combination of good physical condition and insecticidal potency demonstrates nets effectiveness in providing personal protection. The statistical difference between net deterioration and the manufacturer points to underlying factors associated with defects during manufacturing.

Majority of the holes were found on the lower part of the net, with all the sides having almost the same proportions of holes. The main cause of holes on the lower side as reported was due to continuous tucking under the mattress and often, the net getting caught by rough edges. House environment (type of building material), and general handling contribute to bed net deterioration [20]. The uniform rate of deterioration on all the sides of bed nets in this study shows that the social economic status of the population is relatively the same. Deterioration of bed nets have been shown to be higher in poorer communities [4].

More than three quarters of nets with at least one hole had not been repaired, as was the case in an Ethiopian study where very few households had repaired their nets [21]. When participants were asked why they had not repaired their nets, they reported not to know that a net needed repair. A net becomes less protective with increased number of holes even when treated with an insecticide [5].

The survival rate of the nets was high with the only cause of net loss in the study area being giving away of the nets to family members living far away [23].

When owners of the nets found in their original package were asked what they had been using, they reported to have been using nets given during the previous mass net distribution of 2011. This excess ownership of bed nets was also found by Githinji and others in a study conducted in Western Kenya where more than half of the nets in their original package were provided during free mass net campaign [15]. Free mass net distribution of 2006 made Kenya the country with highest number of net distributed at that time in Africa [24]. Sambe and others reported that net owners keep excess nets in order to replace, once the existing nets in use become old [4].

Only a small proportion of the nets in the current study had been used for other purposes (mis-used) rather than protection. Higher rates of net mis-use had been reported by Mutuku and others in Kwale County, Kenya, where up to 21% of distributed nets had been mis-used [25].

House material is a major contributor to the rate of net deterioration. This could possibly explain the cause of holes found on the upper part of the nets, explained as challenges faced during hanging. The net ability to offer protection from mosquito bite could have been compromised if the number of holes on the upper part of the net was big; since it has been shown that mosquitoes are more likely to enter bed nets from the upper part [26].

A safety measure considered during bed net manufacture ensures that if a spark lands on a net the burn will not exceed a few centimetres [6]. This could possibly explain why the holes caused by fire burn, were all size one holes.

There were concentration difference in-between baseline nets which could be attributed to a problem in the manufacturing process. During manufacture cooling and stretching of the polymer segregates permethrin to the fibre surface [27]. This could be responsible for the low initial surface concentration in some parts of the net. This low surface concentration on the surface fibres of a net has also been reported [28].

Abrasion and frequency of use are some of the factors responsible for decreased insecticide concentration within the nets. Some of the nets were found drying outside after a wash on the survey date. This could have been a cause of low surface concentration. A complete regeneration of insecticide from the sub surface of the net to the surface fibres requires approximately 2 weeks at 30^0^ C after washing [29].

Abrasion lowers insecticidal concentration on the surface of a net [13]. This is likely to happen when tucking the net or when the net is being rolled up in the morning [7]. The findings on the net chemical residue are in line with most programmes where a net insecticidal activity of up to 0.00273 mg of permethrin is effective in mosquito control. In a phase III evaluation study of LLINs, it was found that concentration as low as 1.3 mg/m^2^ of α-cypermethrin remaining in a net is effective in killing mosquitoes [30].

Analysis of variance showed that a net chemical content is relatively the same for nets with different number of washes. A washed net would protect an individual just like an unwashed net. Every time a net is washed it loses some of its insecticidal content on the surface fibres, but a replacement of the washed insecticide from the sub-surface to the surface fibres occurs which makes a bed net to be effective throughout its life span of 3 years [31]. The concentrations obtained through GCMS could be low since regeneration process is a continuous process and a time of up to 3 weeks (after washing) is needed for a complete regeneration [32].

## Conclusion

The study focused on the performance of nets under operational conditions in Kirinyaga County by checking integrity, use and care post mass net distribution. After 18 months of field use, more than three-quarters of the nets distributed had survived but integrity had dropped quicker than expected. The slow migration of the insecticide from the sub-surface to the surface could be responsible for the low GCMS results. Standard procedure for field evaluation of surface insecticidal content available to a mosquito after landing on a bed net to rest is recommended.

## Supplementary Information

**Additional file 1.** Informed consent form

**Additional file 2.** Questionnaire

**Additional file 3.** Extraction efficiency chromatogram 1

**Additional file 4.** Extraction efficiency chromatogram 2

**Additional file 5.** Extraction efficiency chromatogram 3

**Additional file 6.** Extraction efficiency chromatogram 4

**Additional file 7.** Extraction efficiency chromatogram 5

**Additional file 8.** Repeatability testing chromatogram 1

**Additional file 9.** Repeatability testing chromatogram 2

**Additional file 10.** Repeatability testing chromatogram 3

**Additional file 11.** Calibration curve

**Additional file 12.** Alphacypermethrin Sample analysis

**Additional file 13.** Permethrin Sample analysis

**Additional file 14.** Ethical approval

## Data Availability

The datasets generated and analysed during the current study are available in the figshare repository, https://figshare.com/search.

## Abbreviations

KEMRI: Kenya Medical Research Institute
LLlNs: Long lasting insecticides nets
WHO: World Health Organization
GC-MS: Gas chromatography mass spectrometer
CDC: Centre for disease control
HPLC: High-performance liquid chromatography
pHI: Proportionate Hole Index
PTFE: Polytetrafluoroethylene
EI: Electron ionisation
SIM: Single ion Monitoring
JKUAT: Jomo Kenyatta University of Agriculture and Technology
CI: Confidence interval
NMCP: National malaria control program
ANOVA: Analysis of variance
SD: Standard deviation
SPSS: Statistical package for social sciences
EDA: Exploratory data analysis
IQR: Inter-quartile range
AI: Active ingredient
N: Number
CHV: Community health volunteers
