# Supplementary material for: Integrity, use and care of long-lasting insecticidal nets in Kirinyaga County, Kenya
Source: BMC Public Health. 2021 May 3;21:856. doi: 10.1186/s12889-021-10882-x (PMC8091527; doi:10.1186/s12889-021-10882-x)
Supplement: Supplementary file 1 — Additional file 1. Informed consent form [file 12889_2021_10882_MOESM1_ESM.docx]

**Additional file 1: Informed consent form**

**PROJECT TITLE: A HOLISTIC ASSESSMENT OF MALARIA IN KIRINYAGA COUNTY: EVALUATION OF ARTEMISININ BASED DRUG RESISTANCE, QUALITY OF THE DRUGS, INSECTICIDE-TREATED NETS AND IMPROVEMENT OF DIAGNOSIS**

**INTRODUCTION**: We are a research team from the Kenya Medical Research Institute (KEMRI). KEMRI is an institution funded by the Government of Kenya to conduct research on human health with a mission to improve it and reduce disease burden on humans. We plan to conduct a study to evaluate the insecticides treated bed nets used in the selected counties. This study has been approved by the KEMRI Scientific Ethical Review Unit (SERU). We would like to seek your permission to participate in the study.

**PURPOSE OF STUDY:** To assess the quality of insecticides treated bed nets if they are still effective in stopping mosquitoes form biting you at night to prevent transmission of malaria. The entire study will last for approximately 2 years. Used bed nets will be randomly selected from households to be used in evaluating the insecticides used in their treatment.

**BENEFITS:** You will not benefit directly from giving us your bed net, but by participating you may contribute towards the fight against malaria in your county and the country as a whole. You will receive a new bed net to replace the one collected from you.

**STORAGE, TRANSPORTATION OF SAMPLES AND FURTHER STUDIES:** The collected bed nets will be transported to KEMRI malaria lab in Nairobi for further studies.

I confirm that I have understood the purpose of this exercise and wish to participate in this research being conducted by the Malaria and Chemistry Units of KEMRI, Nairobi Kenya. I understand that I am free to ask any questions or to withdraw from participation at any time without penalty.

The researcher conducting this study is Ruth Nyangacha. You may ask any questions you have now, or if you have any questions later, you are encouraged to contact her through mobile telephone number: 0728710650 or email her at [rnyangacha@kemri.org](mailto:rnyangacha@kemri.org)

If you have any questions or concerns regarding the study and would like to talk to someone other than the researcher (s), you are encouraged to contact the following:

Name of participant ___________________Signature _____________Date______________

Name of Researcher_________________ Signature ____________Date______________
