# Supplementary material for: Integrity, use and care of long-lasting insecticidal nets in Kirinyaga County, Kenya
Source: BMC Public Health. 2021 May 3;21:856. doi: 10.1186/s12889-021-10882-x (PMC8091527; doi:10.1186/s12889-021-10882-x)
Supplement: Supplementary file 2 — Additional file 2. Questionnaire [file 12889_2021_10882_MOESM2_ESM.docx]

Additional file 2: Questionnaire

| 1 | Serial Number |  |
| --- | --- | --- |
| 2 | Sample code |  |
| 3 | Sampling Date |  |
| 4 | Physical location | 1.Sub-county |
| 5 |  | 2. Location |
| 6 |  | 3.Village |
| 7 | Gender |  |
| 8 | Age of respondent |  |
| 9 | Education level |  |
| 10 | Do you have the nets supplied in 2016 |  |
| 11 | Was it in use last night |  |
| 12 | If no why |  |
| 13 | Date of manufacture |  |
| 14 | Manufacturer’s name |  |
| 15 | Distributer |  |
| 16 | Date of expiry |  |
| 17 | Active pharmaceutical ingredient (API) |  |
| 18 | Approximate number of washes |  |
| 19 | Have you replaced the net? |  |
| 20 | If yes why? |  |
| 21 | What informed replacement? |  |
| 22 | Physical status of the nets |  |
| 23 | - Is the net intact? |  |
| 24 | - Number of holes |  |
| 25 | - Size of holes |  |
| 26 | Has the net been repaired? |  |
| 27 | If yes type of repair |  |
| 28 | Cause of holes |  |
