# Supplementary material for: Integrity, use and care of long-lasting insecticidal nets in Kirinyaga County, Kenya
Source: BMC Public Health. 2021 May 3;21:856. doi: 10.1186/s12889-021-10882-x (PMC8091527; doi:10.1186/s12889-021-10882-x)
Supplement: Supplementary file 5 — Additional file 5. Extraction efficiency chromatogram 3 [file 12889_2021_10882_MOESM5_ESM.pdf]

Sample Information

Analyzed by	: Admin
Analyzed	: 6/26/2019 3:19:03 PM
Sample Type	: Unknown
Level #	: 1
Sample Name	: 190625_mary_BL3
Sample ID	: STD-0034
IS Amount	: [1]=1
Sample Amount	: 1
Dilution Factor	: 1
Vial #	: 34
Injection Volume	: 1.00
Data File	: C:\GCMSsolution\Data\Project1\Martin\Mary_KEMRI_1\190625_mary_BL3.qgd
Org Data File	: C:\GCMSsolution\Data\Project1\Martin\Mary_KEMRI_1\190625_mary_BL3.qgd
Method File	: C:\GCMSsolution\Data\Project1\Martin\Mary_KEMRI_1\Pesticides_mary_quant.qgm
Org Method File	: C:\GCMSsolution\Data\Project1\Martin\Pesticides_mary.qgm
Report File	:
Tuning File	: C:\GCMSsolution\System\Tune1\Default.qgt
Modified by	: Admin
Modified	: 8/8/2019 12:17:43 PM

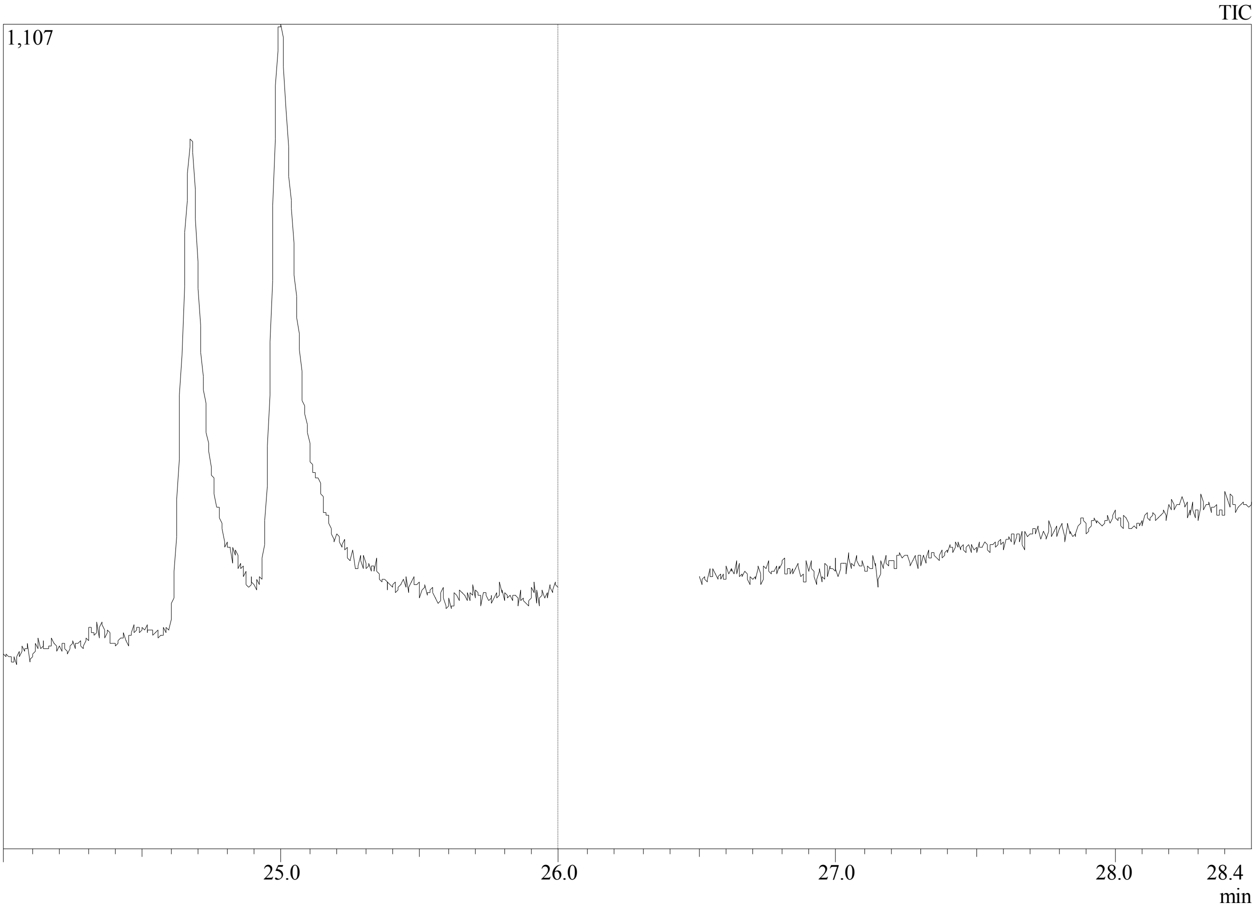

Library

No peaks found
