# Supplementary material for: Integrity, use and care of long-lasting insecticidal nets in Kirinyaga County, Kenya
Source: BMC Public Health. 2021 May 3;21:856. doi: 10.1186/s12889-021-10882-x (PMC8091527; doi:10.1186/s12889-021-10882-x)

C:\GCMSsolution\Data\Project1\13082019_mary_500ppb_rep001.qgd

Quantitative Result Table

ID#	R.Time	m/z	Area	Height	Conc.	Name
1	24.65	183.00	1352	303	143.27 ppb	permethrin
2	27.17	163.00	150	47	79.86 ppb	cypermethrin

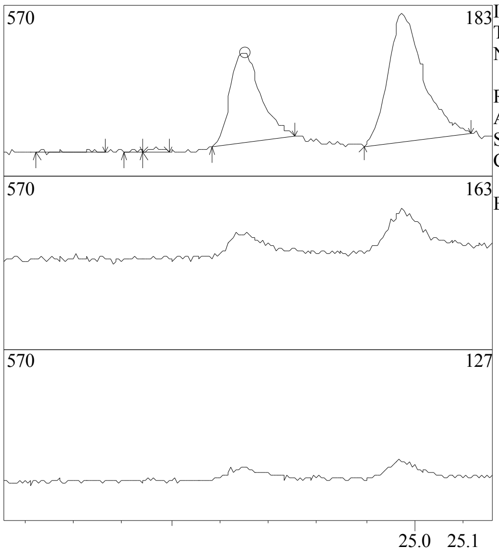

Quantitation

ID#:1 Mass:183.00
Type:Target
Name:permethrin

R.T:24.653
Area:1352
SI:99
Conc:143.26509ppb

Event:1:SIM

#	m/z	Intensity	Ratio
1	163.00	70	23.10
2	127.00	38	12.54

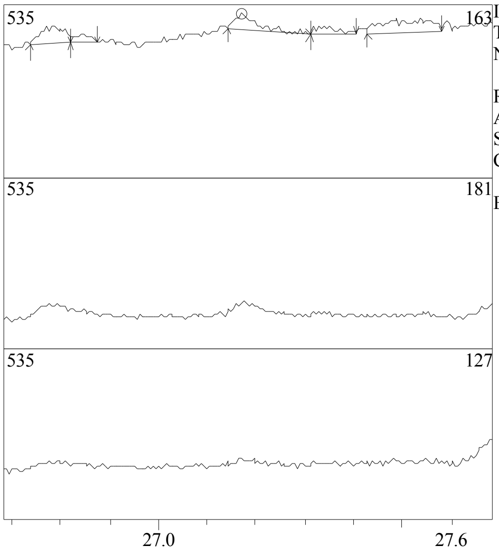

ID#:2 Mass:163.00
Type:Target
Name:cypermethrin

R.T:27.171
Area:150
SI:96
Conc:79.85650ppb

Event:1:SIM

#	m/z	Intensity	Ratio
1	181.00	25	64.10
2	127.00	7	17.95

Chromatogram 13082019_mary_500ppb_rep C:\GCMSsolution\Data\Project1\13082019_mary_500ppb_rep001.qgd

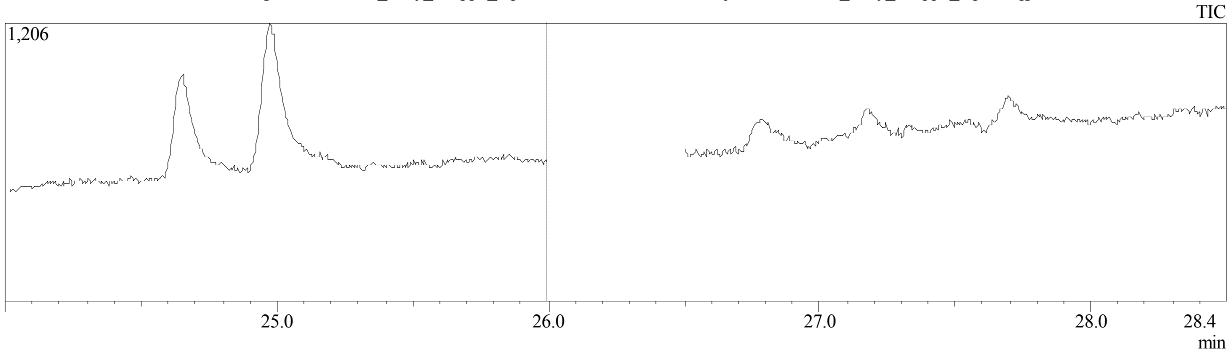

Supplement: Supplementary file 8 — Additional file 8. Repeatability testing chromatogram 1 [file 12889_2021_10882_MOESM8_ESM.pdf]
