# Supplementary material for: Integrity, use and care of long-lasting insecticidal nets in Kirinyaga County, Kenya
Source: BMC Public Health. 2021 May 3;21:856. doi: 10.1186/s12889-021-10882-x (PMC8091527; doi:10.1186/s12889-021-10882-x)
Supplement: Supplementary file 9 — Additional file 9. Repeatability testing chromatogram 2 [file 12889_2021_10882_MOESM9_ESM.pdf]

Sample Information

Analyzed by	: Admin
Analyzed	: 8/19/2019 5:07:36 PM
Sample Type	: Unknown
Level #	: 1
Sample Name	: 19082019_mary_rep
Sample ID	: STD-0002
IS Amount	: [1]=1
Sample Amount	: 1
Dilution Factor	: 1
Vial #	: 1
Injection Volume	: 1.00
Data File	: C:\GCMSsolution\Data\Project1\19082019_mary_rep001.qgd
Org Data File	: C:\GCMSsolution\Data\Project1\19082019_mary_rep001.qgd
Method File	: C:\GCMSsolution\Data\Project1\Martin\Mary_KEMRI_1\Pesticides_mary_quant.qgm
Org Method File	: C:\GCMSsolution\Data\Project1\Martin\Pesticides_mary.qgm
Report File	:
Tuning File	: C:\GCMSsolution\System\Tune1\Default.qgt
Modified by	: Admin
Modified	: 9/27/2019 3:48:25 PM

Quantitative Result Table

ID#	Name	Conc.	Conc.Unit	R.Time	m/z	Area	Height
1	permethrin	443.176	ppb	24.652	183.00	5141	1202
2	cypermethrin	135.412	ppb	27.185	163.00	650	158

Chromatogram 19082019_mary_rep C:\GCMSsolution\Data\Project1\19082019_mary_rep001.qgd

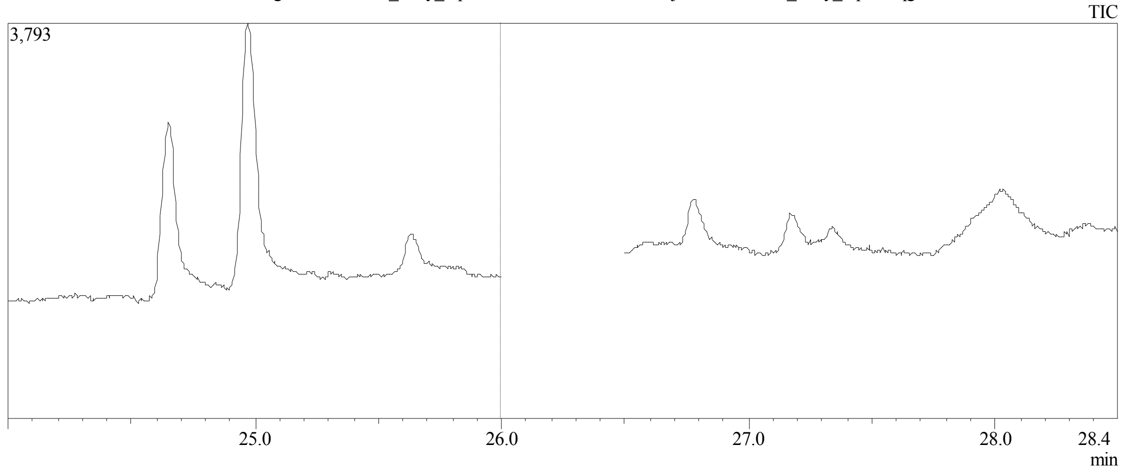

Quantitation

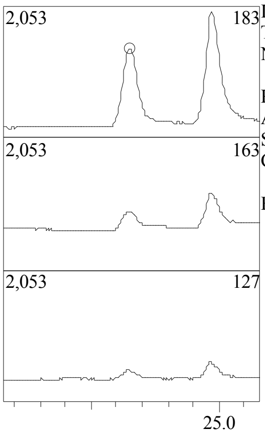

ID#:1 Mass:183.00
Type:Target
Name:permethrin

R.T:24.652
Area:5141
SI:99
Conc:443.17593ppb

Event:1:SIM

#	m/z	Intensity	Ratio
1	163.00	295	24.73
2	127.00	133	11.15

Calibration

ID#:1 Mass:183.00 Name:permethrin
f(x)=12.633755*x-457.976103
rr1=0.999127 rr2=0.998255
MeanRF:10.94 RFSD:1.43 RFRSD:13.08
CurveType:Least Square Method
ZeroThrough:Not Through
WeightedRegression:1/C2
External Standard

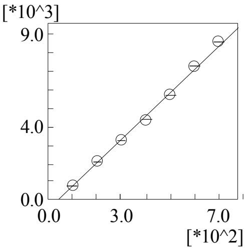

#	Conc. (ppb)	Mean Area
1	100.000	804.00
2	200.000	2130.00
3	300.000	3284.00
4	400.000	4366.00
5	500.000	5752.00
6	600.000	7297.00
7	700.000	8641.00

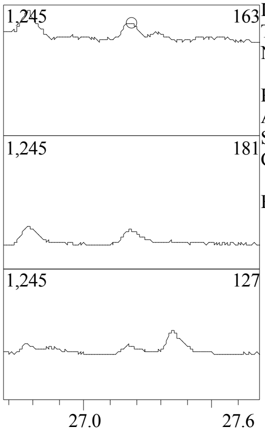

ID#:2 Mass:163.00
Type:Target
Name:cypermethrin

R.T:27.185
Area:650
SI:98
Conc:135.41244ppb

Event:1:SIM

#	m/z	Intensity	Ratio
1	181.00	102	75.00
2	127.00	48	35.29

ID#:2 Mass:163.00 Name:cypermethrin
f(x)=8.999937*x-568.703460
rr1=0.999000 rr2=0.998001
MeanRF:6.75 RFSD:1.86 RFRSD:27.63
CurveType:Least Square Method
ZeroThrough:Not Through
WeightedRegression:1/C2
External Standard

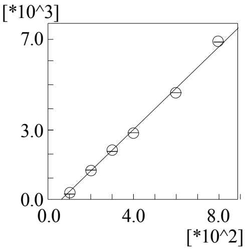

#	Conc. (ppb)	Mean Area
1	100.000	324.00
2	200.000	1277.00
3	300.000	2172.00
4	400.000	2910.00
6	600.000	4654.00
8	800.000	6877.00
