# Supplementary material for: Integrity, use and care of long-lasting insecticidal nets in Kirinyaga County, Kenya
Source: BMC Public Health. 2021 May 3;21:856. doi: 10.1186/s12889-021-10882-x (PMC8091527; doi:10.1186/s12889-021-10882-x)

C:\GCMSsolution\Data\Project1\16082019_mary_rep001.qgd

Quantitative Result Table

ID#	R.Time	m/z	Area	Height	Conc.	Name
1	24.64	183.00	1672	398	168.59 ppb	permethrin
2	27.16	163.00	438	79	111.86 ppb	cypermethrin

Quantitation

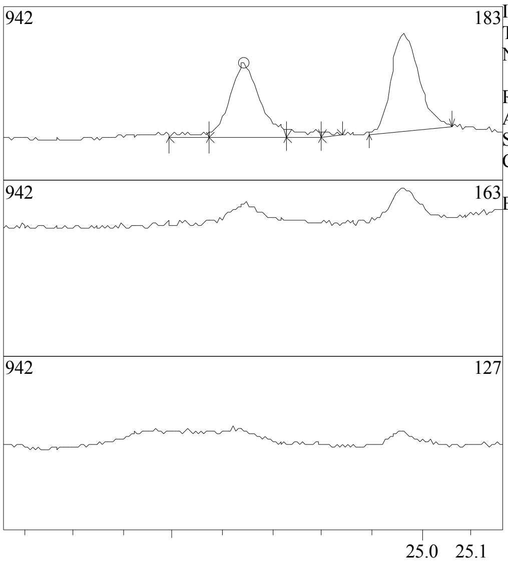

ID#:1 Mass:183.00
Type:Target
Name:permethrin

R.T:24.643
Area:1672
SI:99
Conc:168.59406ppb

Event:1:SIM

#	m/z	Intensity	Ratio
1	163.00	95	26.24
2	127.00	32	8.84

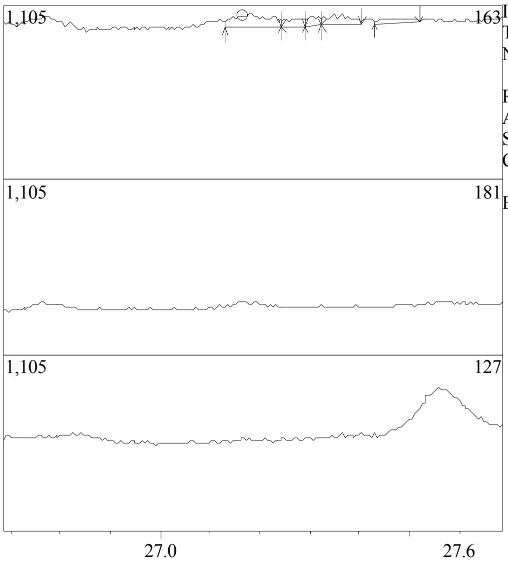

ID#:2 Mass:163.00
Type:Target
Name:cypermethrin

R.T:27.164
Area:438
SI:95
Conc:111.85672ppb

Event:1:SIM

#	m/z	Intensity	Ratio
1	181.00	30	93.75
2	127.00	9	28.13

Chromatogram 16082019_mary_rep C:\GCMSsolution\Data\Project1\16082019_mary_rep001.qgd

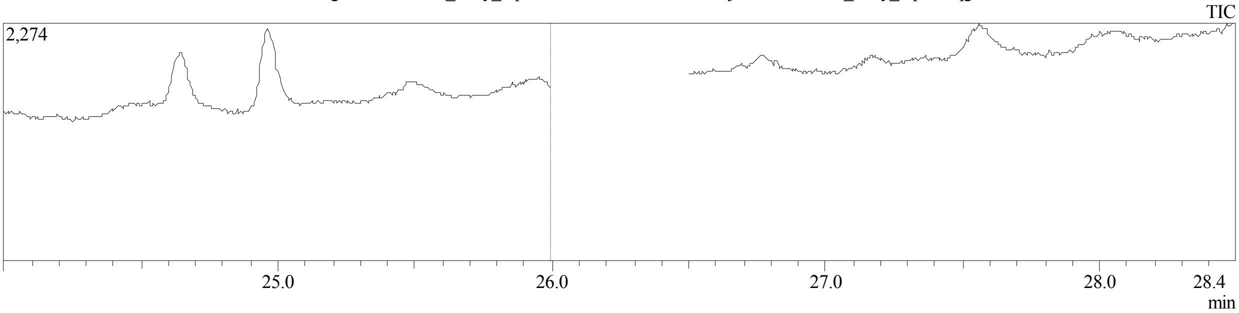

Supplement: Supplementary file 10 — Additional file 10. Repeatability testing chromatogram 3 [file 12889_2021_10882_MOESM10_ESM.pdf]
