# Supplementary material for: Integrity, use and care of long-lasting insecticidal nets in Kirinyaga County, Kenya
Source: BMC Public Health. 2021 May 3;21:856. doi: 10.1186/s12889-021-10882-x (PMC8091527; doi:10.1186/s12889-021-10882-x)
Supplement: Supplementary file 11 — Additional file 11. Calibration curve [file 12889_2021_10882_MOESM11_ESM.pdf]

Sample Information

Analyzed by	: Admin
Analyzed	: 6/26/2019 1:25:45 AM
Sample Type	: Unknown
Level #	: 1
Sample Name	: 190625_mary_24
Sample ID	: STD-0013
IS Amount	: [1]=1
Sample Amount	: 1
Dilution Factor	: 1
Vial #	: 13
Injection Volume	: 1.00
Data File	: C:\GCMSsolution\Data\Project1\Martin\Mary_KEMRI_1\190625_mary_24.qgd
Org Data File	: C:\GCMSsolution\Data\Project1\Martin\Mary_KEMRI_1\190625_mary_24.qgd
Method File	: C:\GCMSsolution\Data\Project1\Martin\Mary_KEMRI_1\Pesticides_mary_quant.qgm
Org Method File	: C:\GCMSsolution\Data\Project1\Martin\Pesticides_mary.qgm
Report File	:
Tuning File	: C:\GCMSsolution\System\Tune1\Default.qgt
Modified by	: Admin
Modified	: 9/27/2019 1:49:24 PM

Quantitative Result Table

ID#	Name	Conc.	Conc.Unit	R.Time	m/z	Area	Height
1	permethrin	257.592	ppb	24.673	183.00	2545	508
2	cypermethrin	N.D.(Ref)	ppb	-	163.00	---	---

Chromatogram 190625_mary_24 C:\GCMSsolution\Data\Project1\Martin\Mary_KEMRI_1\190625_mary_24.qgd

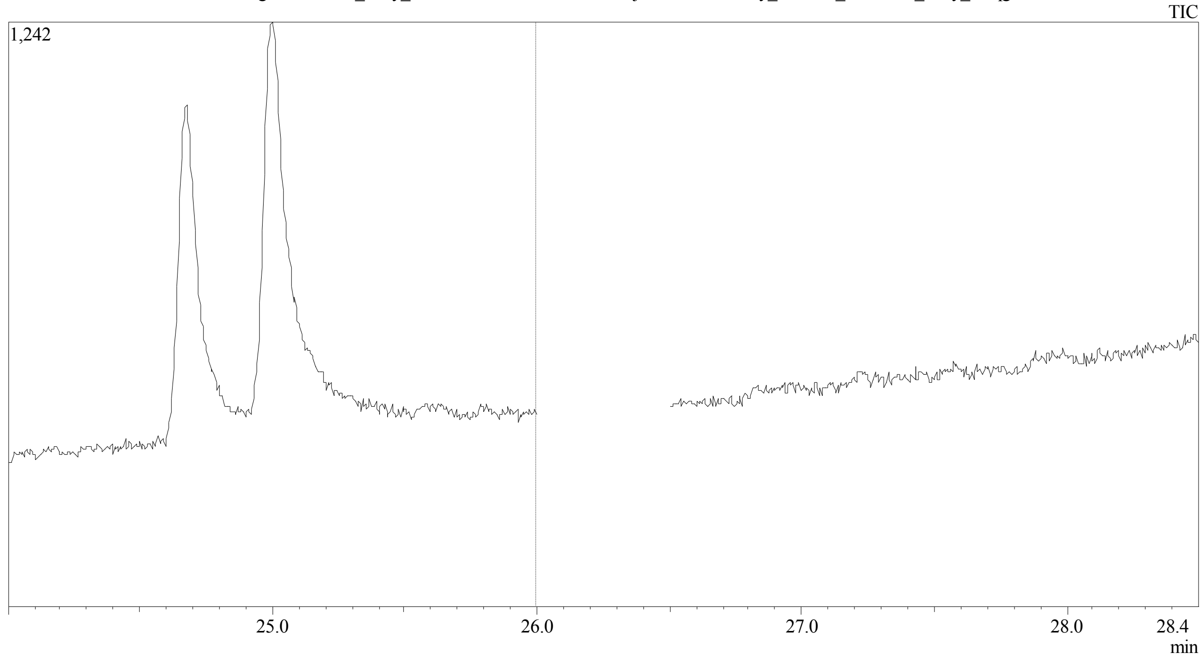

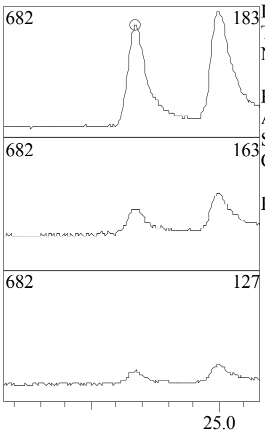

Quantitation

ID#:1 Mass:183.00
Type:Target
Name:permethrin

R.T:24.673
Area:2545
SI:99
Conc:257.59248ppb

Event:1:SIM

#	m/z	Intensity	Ratio
1	163.00	117	24.48
2	127.00	54	11.30

Calibration

ID#:1 Mass:183.00 Name:permethrin
f(x)=14.892177*x-1291.112903
rr1=0.977152 rr2=0.954827
MeanRF:11.49 RFSD:1.96 RFRSD:17.04
CurveType:Least Square Method
ZeroThrough:Not Through
WeightedRegression:None
External Standard

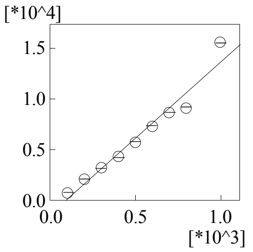

#	Conc. (ppb)	Mean Area
1	100.000	804.00
2	200.000	2130.00
3	300.000	3284.00
4	400.000	4366.00
5	500.000	5752.00
6	600.000	7297.00
7	700.000	8641.00
8	800.000	9089.00
9	1000.000	15521.00

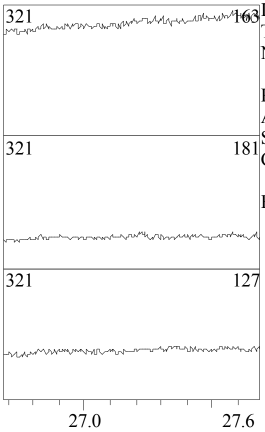

ID#:2 Mass:163.00
Type:Target
Name:cypermethrin

R.T:27.275
Area:124
SI:91
Conc: N.D.(Ref)

Event:1:SIM

#	m/z	Intensity	Ratio
1	181.00	3 *	25.00
2	127.00	3	25.00

ID#:2 Mass:163.00 Name:cypermethrin
f(x)=11.304177*x-1258.912903
rr1=0.981354 rr2=0.963056
MeanRF:7.80 RFSD:2.28 RFRSD:29.21
CurveType:Least Square Method
ZeroThrough:Not Through
WeightedRegression:None
External Standard

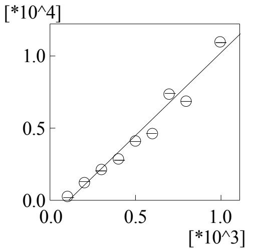

#	Conc. (ppb)	Mean Area
1	100.000	324.00
2	200.000	1277.00
3	300.000	2172.00
4	400.000	2910.00
5	500.000	4139.00
6	600.000	4654.00
7	700.000	7364.00
8	800.000	6877.00
9	1000.000	10952.00
