# Supplementary material for: Integrity, use and care of long-lasting insecticidal nets in Kirinyaga County, Kenya
Source: BMC Public Health. 2021 May 3;21:856. doi: 10.1186/s12889-021-10882-x (PMC8091527; doi:10.1186/s12889-021-10882-x)
Supplement: Supplementary file 12 — Additional file 12. Alphacypermethrin Sample analysis [file 12889_2021_10882_MOESM12_ESM.doc]

Sample analysis for α-cypermethrin treated samples

| **Sample Name** | **Quantifier Ion** | **Permethrin** | | **α-cypermethrin** | |
| --- | --- | --- | --- | --- | --- |
|  |  | Area | Instrument conc. in ppb | Area | Instrument conc. in ppb |
| **64** | 163 m/z | - | - | 71 | 71.08 |
| **65** | 163 m/z |  |  |  |  |
| **66** | 163 m/z | - | - | 415 | 109.30 |
| **67** | 163 m/z | - | - | 189 | 84.19 |
| **68** | 163 m/z | 4226 | 370.75 | - | - |
| **69** | 163 m/z | - | - | - | - |
| **70** | 163 m/z | - | - | 433 | 111.30 |
| **71** | 163 m/z | - | - | 174 | 82.52 |
| **72** | 163 m/z | - | - | - | - |
| **73** | 163 m/z | - | - | 152 | 80.08 |
| **74** | 163 m/z | - | - | - | - |
| **75** | 163 m/z | - | - | 86 | 72.75 |
| **76** | 163 m/z |  |  |  |  |
| **77** | 163 m/z | 950 | 111.45 | 1883 | 272.41 |
| **78** | 163 m/z | 1198 | 131.08 | 850 | 157.63 |
| **79** | 163 m/z | 1233 | 133.85 | 1137 | 189.52 |
| **80** | 163 m/z |  |  |  |  |
| **81** | 163 m/z | - | - | 401 | 107.75 |
| **82** | 163 m/z | 486 | 74.72 | 2137 | 300.64 |
| **83** | 163 m/z | 32869 | 2637.93 | - | - |
| **84** | 163 m/z | 3009 | 274.42 | 1709 | 253.08 |
| **85** | 163 m/z | 18989 | 1539.29 | 359 | 103.08 |
| **86** | 163 m/z | 12857 | 1053.92 | 896 | 162.75 |
| **87** | 163 m/z | - | - | - | - |
| **88** | 163 m/z | 762 | 95.56 | 433 | 111.30 |
| **89** | 163 m/z | 14868 | 1213.10 | - | - |
| **90** | 163 m/z | - | - | 908 | 164.08 |
| **91** | 163 m/z | - | - | 1361 | 214.41 |
| **92** | 163 m/z | 391 | 67.20 | 1087 | 183.97 |
| **93** | 163 m/z | - | - | 233 | 89.08 |
| **94** | 163 m/z | 524 | 77.73 | 2250 | 313.19 |
| **95** | 163 m/z | - | - | 83 | 72.41 |
| **96** | 163 m/z | 436 | 70.76 | 3058 | 402.97 |
| **97** | 163 m/z | 132 | 46.70 | 3058 | 402.97 |
| **98** | 163 m/z | 60 | 44.00 | - | - |
| **99** | 163 m/z | - | - | 943 | 167.97 |
| **100** | 163 m/z | 15356 | 1251.72 | 658 | 136.30 |
| **101** | 163 m/z | - | - | 384 | 105.86 |
| **102** | 163 m/z | 127 | 46.30 | 816 | 153.86 |
| **103** | 163 m/z | 296 | 59.68 | 2671 | 359.97 |
