# Supplementary material for: Integrity, use and care of long-lasting insecticidal nets in Kirinyaga County, Kenya
Source: BMC Public Health. 2021 May 3;21:856. doi: 10.1186/s12889-021-10882-x (PMC8091527; doi:10.1186/s12889-021-10882-x)
Supplement: Supplementary file 13 — Additional file 13. Permethrin Sample analysis [file 12889_2021_10882_MOESM13_ESM.doc]

Sample analysis for permethrin treated samples

| **Sample name** | **Quantifier ion** | **Permethrin** | | **α-cypermethrin** | |
| --- | --- | --- | --- | --- | --- |
|  |  | Area | Instrument conc. in ppb | Area | Instrument conc. in ppb |
| **23** | 183 m/z | - | - | - | - |
| **24** | 183 m/z | 2545 | 257.50 | - | - |
| **25** | 183 m/z | - | - | - | - |
| **26** | 183 m/z | 304 | 107.11 | 26 | 113.67 |
| **27** | 183 m/z | - | - | - | - |
| **28** | 183 m/z | 846 | 143.51 | - | - |
| **29** | 183 m/z | 1572 | 192.26 | - | - |
| **30** | 183 m/z | - |  |  |  |
| **31** | 183 m/z | 347 | 63.72 | 60 | 69.86 |
| **32** | 183 m/z | - | - | - | - |
| **33** | 183 m/z | - | - | - | - |
| **34** | 183 m/z | 1779 | 177.06 | - | - |
| **35** | 183 m/z | - | - | - | - |
| **36** | 183 m/z | - | - | 29 | 113.93 |
| **37** | 183 m/z | 299 | 59.92 | - | - |
| **38** | 183 m/z | 114 | 94.35 | - | - |
| **39** | 183 m/z | 367 | 111.34 | - | - |
| **40** | 183 m/z | 284 | 58.73 | - | - |
| **41** | 183 m/z | - | - | - |  |
| **42** | 183 m/z | - | - | - | - |
| **43** | 183 m/z | - | - | - | - |
| **44** | 183 m/z | - | - | - | - |
| **45** | 183 m/z |  |  |  |  |
| **46** | 183 m/z | - | - | - | - |
| **47** | 183 m/z | - | - | - | - |
| **48** | 183 m/z | - | - | 29 | 66.41 |
| **49** | 183 m/z | - | - | - | - |
| **50** | 183 m/z | 3187 | 288.51 |  | - |
| **51** | 183 m/z | 411 | 68.78 | - | - |
| **52** | 183 m/z | - | - | - | - |
| **53** | 183 m/z | - | - | - | - |
| **54** | 183 m/z | 365 | 65.14 | - | - |
| **55** | 183 m/z | - | - | - | - |
| **56** | 183 m/z | - | - | - | - |
| **57** | 183 m/z | 93 | 47.81 | - | - |
| **58** | 183 m/z | - | - | - | - |
| **59** | 183 m/z | - | - | - | - |
| **60** | 183 m/z | 618 | 85.17 | - | - |
| **61** | 183 m/z | - | - | 107 | 75.08 |
| **62** | 183 m/z | - | - | - | - |
| **63** | 183 m/z | - | - | - | - |
