# Supplementary figures and images for: Integrity, use and care of long-lasting insecticidal nets in Kirinyaga County, Kenya
Source: BMC Public Health. 2021 May 3;21:856. doi: 10.1186/s12889-021-10882-x (PMC8091527; doi:10.1186/s12889-021-10882-x)

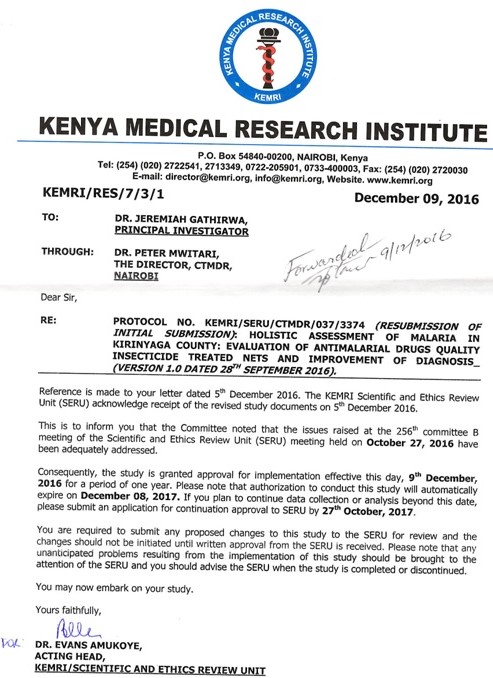

Supplement: Supplementary file 14 — Additional file 14. Ethical approval [file 12889_2021_10882_MOESM14_ESM.doc]
